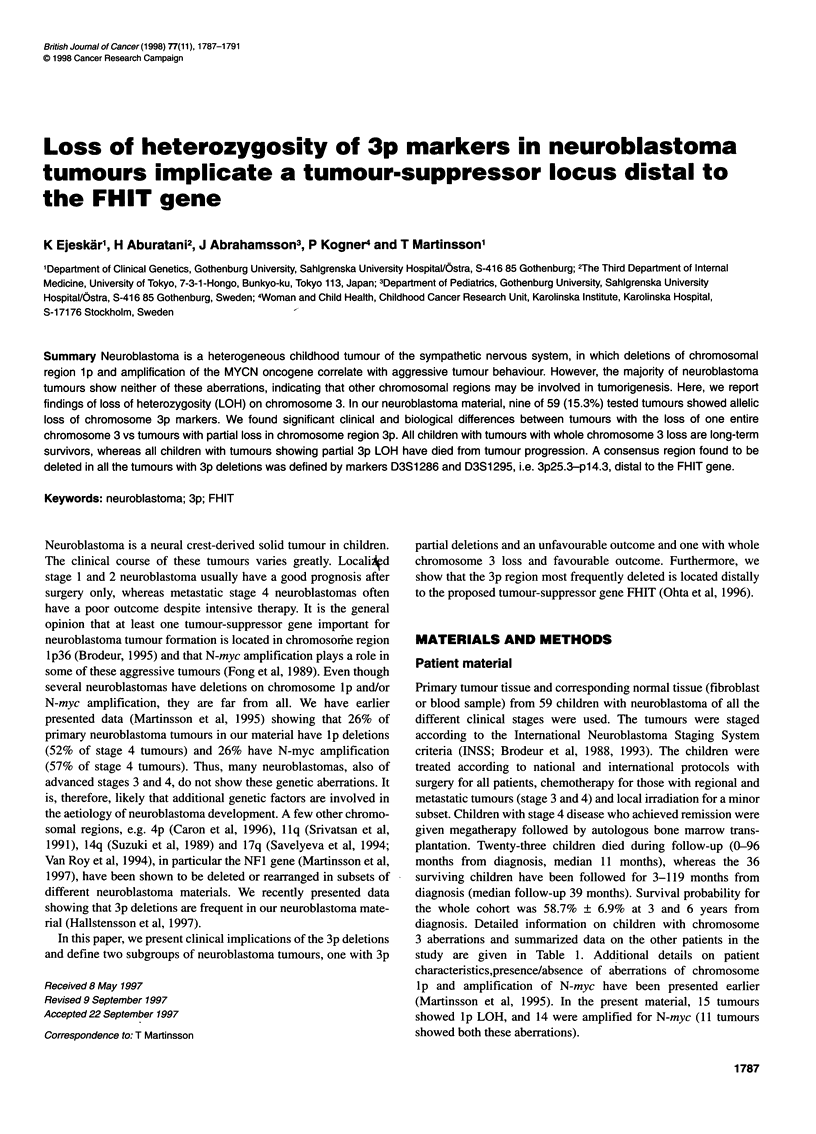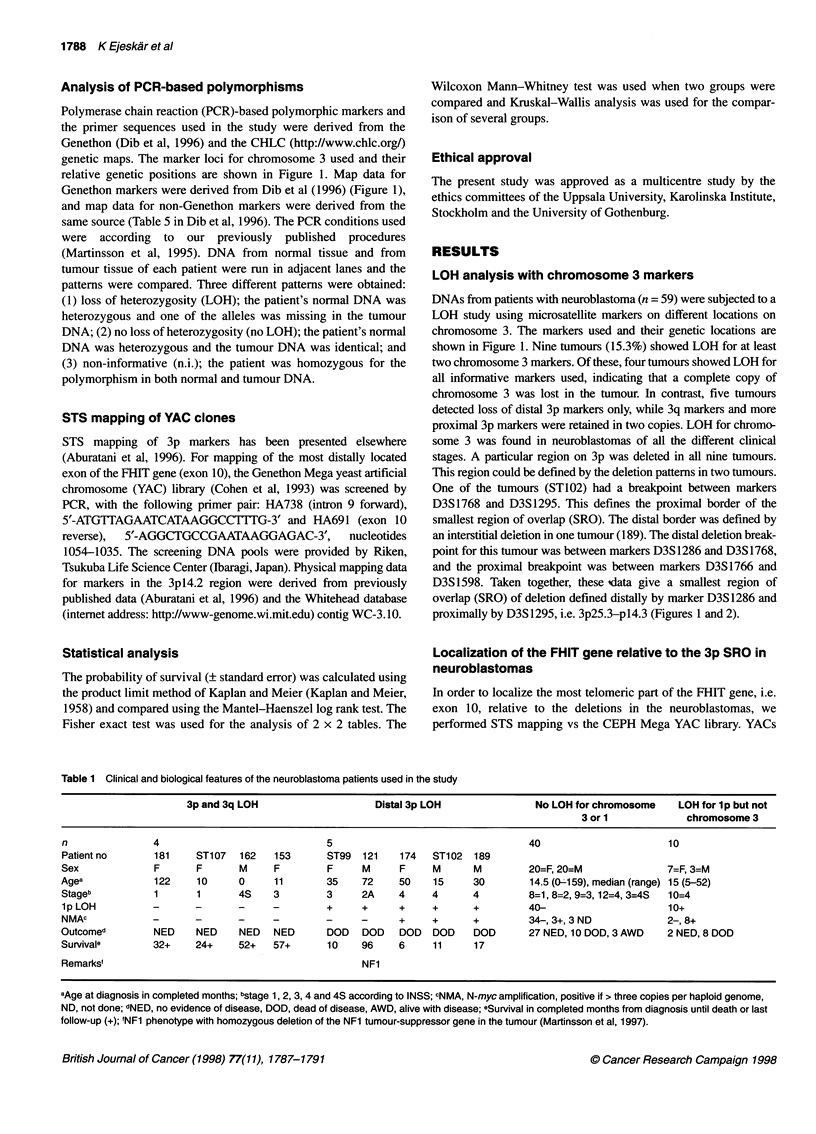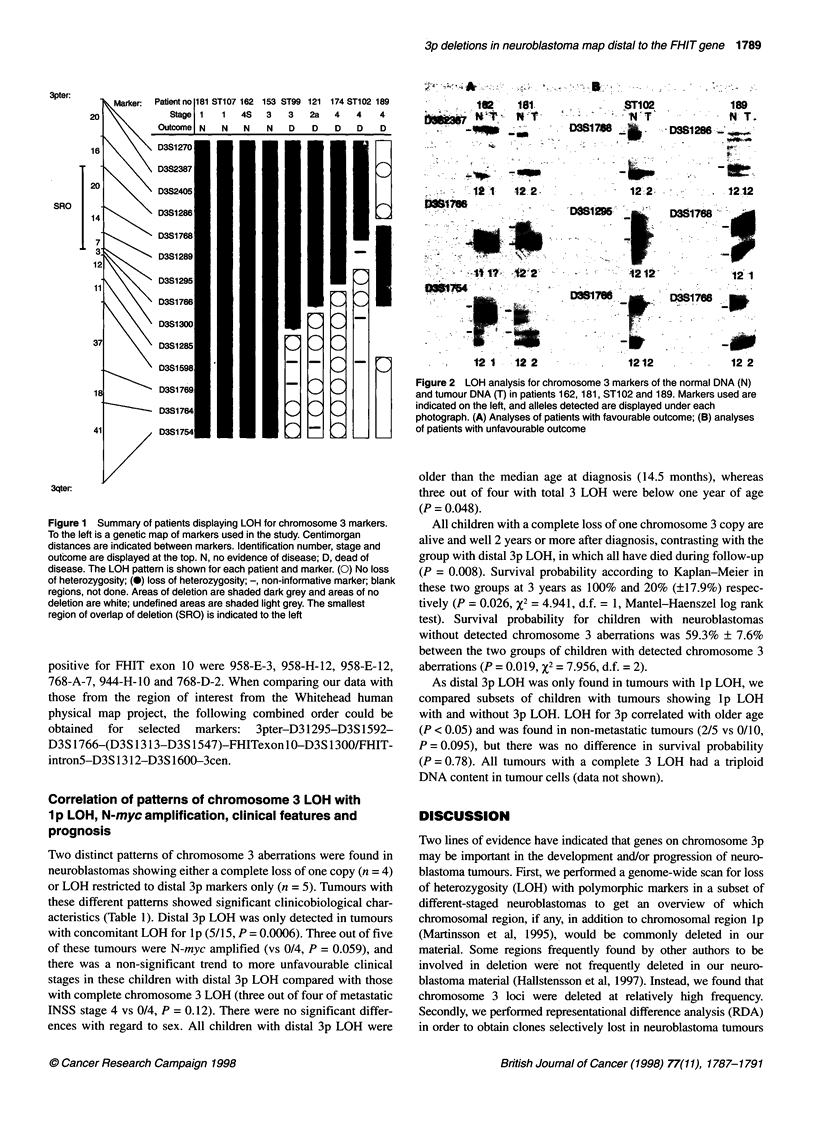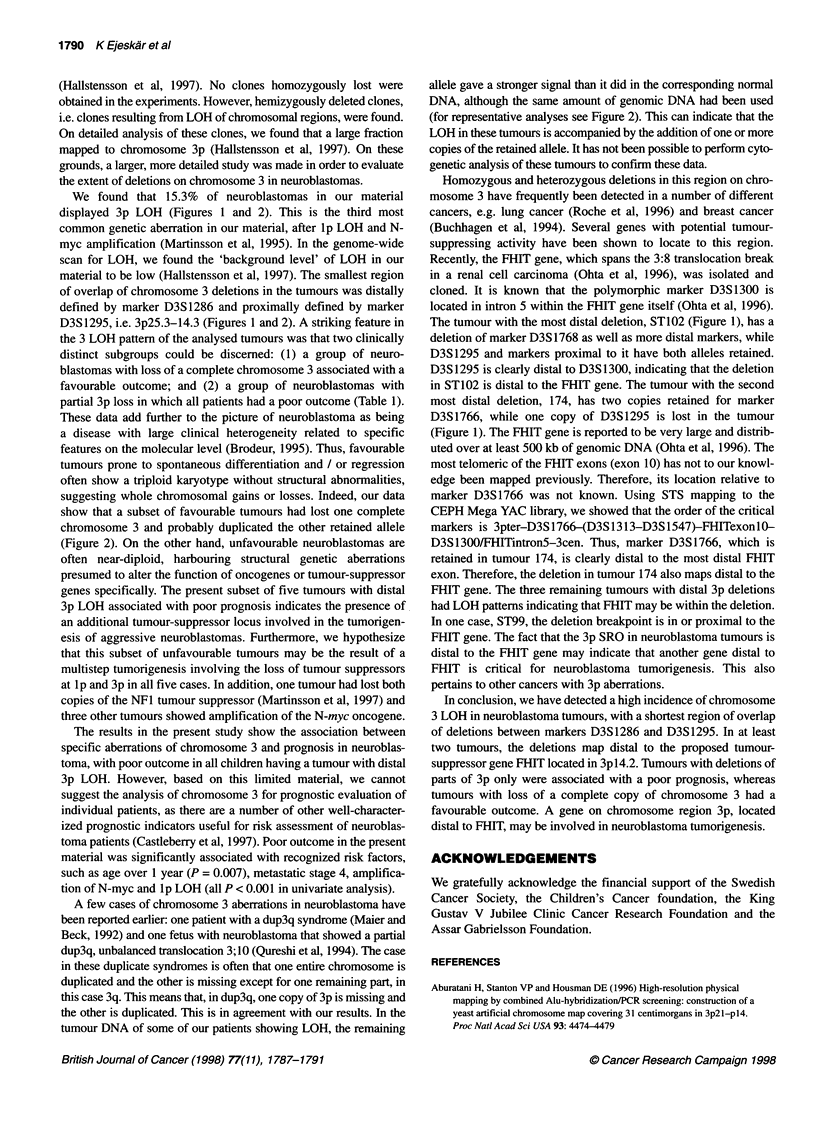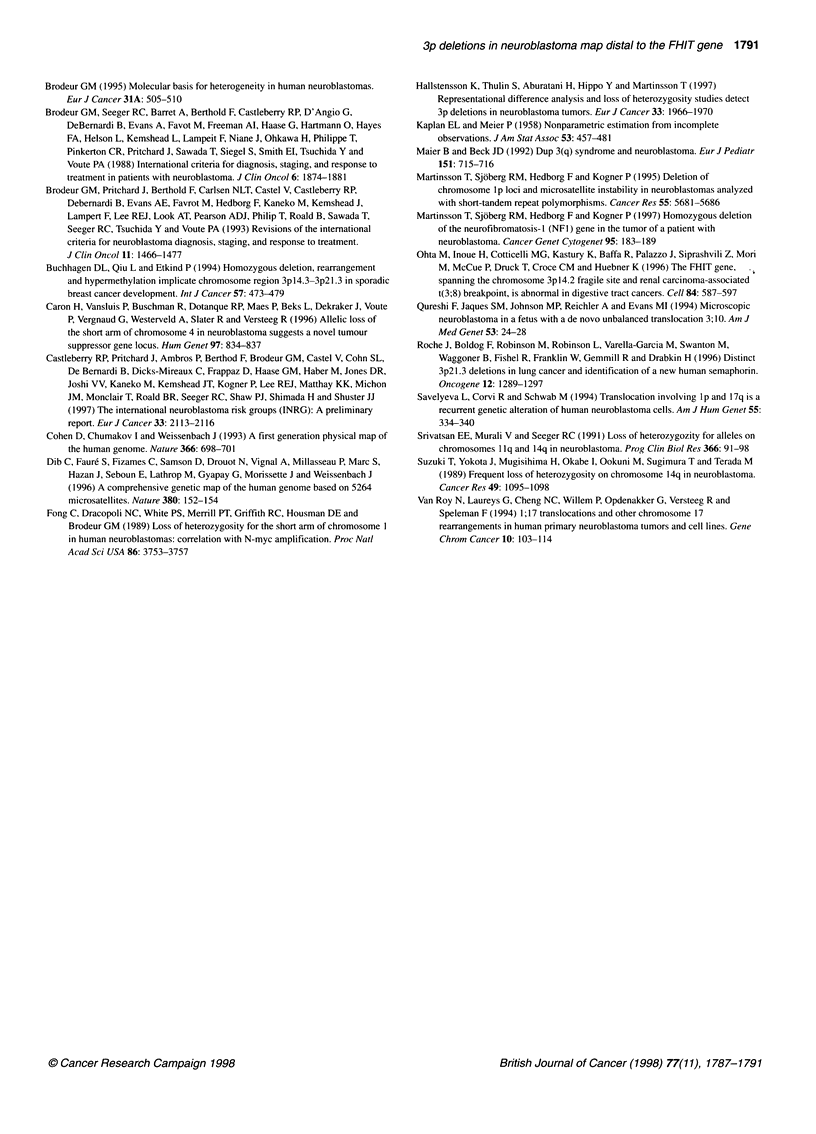# Loss of heterozygosity of 3p markers in neuroblastoma tumours implicate a tumour-suppressor locus distal to the FHIT gene.

**DOI:** 10.1038/bjc.1998.297

**Published:** 1998-06

**Authors:** K. EjeskÃ¤r, H. Aburatani, J. Abrahamsson, P. Kogner, T. Martinsson

**Affiliations:** Department of Clinical Genetics, Gothenburg University, Sahlgrenska University Hospital/Ostra, Sweden.

## Abstract

**Images:**


British Journal of Cancer (1998) 77(11), 1787–1791
© 1998 Cancer Research Campaign

### Loss of heterozygosity of 3p markers in neuroblastoma tumours implicate a tumour-suppressor locus distal to the FHIT gene

**K Ejeskär[1], H Aburatani[2], J Abrahamsson[3], P Kogner[4] and T Martinsson[1]**

[1]Department of Clinical Genetics, Gothenburg University, Sahlgrenska University Hospital/Östra, S-416 85 Gothenburg; [2]The Third Department of Internal Medicine, University of Tokyo, 7-3-1-Hongo, Bunkyo-ku, Tokyo 113, Japan; [3]Department of Pediatrics, Gothenburg University, Sahlgrenska University Hospital/Östra, S-416 85 Gothenburg, Sweden; [4]Woman and Child Health, Childhood Cancer Research Unit, Karolinska Institute, Karolinska Hospital, S-17176 Stockholm, Sweden

**Summary** Neuroblastoma is a heterogeneous childhood tumour of the sympathetic nervous system, in which deletions of chromosomal region 1p and amplification of the MYCN oncogene correlate with aggressive tumour behaviour. However, the majority of neuroblastoma tumours show neither of these aberrations, indicating that other chromosomal regions may be involved in tumorigenesis. Here, we report findings of loss of heterozygosity (LOH) on chromosome 3. In our neuroblastoma material, nine of 59 (15.3%) tested tumours showed allelic loss of chromosome 3p markers. We found significant clinical and biological differences between tumours with the loss of one entire chromosome 3 vs tumours with partial loss in chromosome region 3p. All children with tumours with whole chromosome 3 loss are long-term survivors, whereas all children with tumours showing partial 3p LOH have died from tumour progression. A consensus region found to be deleted in all the tumours with 3p deletions was defined by markers D3S1286 and D3S1295, i.e. 3p25.3–p14.3, distal to the FHIT gene.

**Keywords:** neuroblastoma; 3p; FHIT

Neuroblastoma is a neural crest-derived solid tumour in children. The clinical course of these tumours varies greatly. Localized stage 1 and 2 neuroblastoma usually have a good prognosis after surgery only, whereas metastatic stage 4 neuroblastomas often have a poor outcome despite intensive therapy. It is the general opinion that at least one tumour-suppressor gene important for neuroblastoma tumour formation is located in chromosome region 1p36 (Brodeur, 1995) and that N-*myc* amplification plays a role in some of these aggressive tumours (Fong et al, 1989). Even though several neuroblastomas have deletions on chromosome 1p and/or N-*myc* amplification, they are far from all. We have earlier presented data (Martinsson et al, 1995) showing that 26% of primary neuroblastoma tumours in our material have 1p deletions (52% of stage 4 tumours) and 26% have N-myc amplification (57% of stage 4 tumours). Thus, many neuroblastomas, also of advanced stages 3 and 4, do not show these genetic aberrations. It is, therefore, likely that additional genetic factors are involved in the aetiology of neuroblastoma development. A few other chromosomal regions, e.g. 4p (Caron et al, 1996), 11q (Srivatsan et al, 1991), 14q (Suzuki et al, 1989) and 17q (Savelyeva et al, 1994; Van Roy et al, 1994), in particular the NF1 gene (Martinsson et al, 1997), have been shown to be deleted or rearranged in subsets of different neuroblastoma materials. We recently presented data showing that 3p deletions are frequent in our neuroblastoma material (Hallstensson et al, 1997).

In this paper, we present clinical implications of the 3p deletions and define two subgroups of neuroblastoma tumours, one with 3p partial deletions and an unfavourable outcome and one with whole chromosome 3 loss and favourable outcome. Furthermore, we show that the 3p region most frequently deleted is located distally to the proposed tumour-suppressor gene FHIT (Ohta et al, 1996).

*Received 8 May 1997*
*Revised 9 September 1997*
*Accepted 22 September 1997*

*Correspondence to:* T Martinsson

### MATERIALS AND METHODS

### Patient material

Primary tumour tissue and corresponding normal tissue (fibroblast or blood sample) from 59 children with neuroblastoma of all the different clinical stages were used. The tumours were staged according to the International Neuroblastoma Staging System criteria (INSS; Brodeur et al, 1988, 1993). The children were treated according to national and international protocols with surgery for all patients, chemotherapy for those with regional and metastatic tumours (stage 3 and 4) and local irradiation for a minor subset. Children with stage 4 disease who achieved remission were given megatherapy followed by autologous bone marrow transplantation. Twenty-three children died during follow-up (0–96 months from diagnosis, median 11 months), whereas the 36 surviving children have been followed for 3–119 months from diagnosis (median follow-up 39 months). Survival probability for the whole cohort was 58.7% ± 6.9% at 3 and 6 years from diagnosis. Detailed information on children with chromosome 3 aberrations and summarized data on the other patients in the study are given in Table 1. Additional details on patient characteristics,presence/absence of aberrations of chromosome 1p and amplification of N-*myc* have been presented earlier (Martinsson et al, 1995). In the present material, 15 tumours showed 1p LOH, and 14 were amplified for N-*myc* (11 tumours showed both these aberrations).

### Analysis of PCR-based polymorphisms

Polymerase chain reaction (PCR)-based polymorphic markers and the primer sequences used in the study were derived from the Genethon (Dib et al, 1996) and the CHLC (http://www.chlc.org/) genetic maps. The marker loci for chromosome 3 used and their relative genetic positions are shown in Figure 1. Map data for Genethon markers were derived from Dib et al (1996) (Figure 1), and map data for non-Genethon markers were derived from the same source (Table 5 in Dib et al, 1996). The PCR conditions used were according to our previously published procedures (Martinsson et al, 1995). DNA from normal tissue and from tumour tissue of each patient were run in adjacent lanes and the patterns were compared. Three different patterns were obtained: (1) loss of heterozygosity (LOH); the patient's normal DNA was heterozygous and one of the alleles was missing in the tumour DNA; (2) no loss of heterozygosity (no LOH); the patient's normal DNA was heterozygous and the tumour DNA was identical; and (3) non-informative (n.i.); the patient was homozygous for the polymorphism in both normal and tumour DNA.

### STS mapping of YAC clones

STS mapping of 3p markers has been presented elsewhere (Aburatani et al, 1996). For mapping of the most distally located exon of the FHIT gene (exon 10), the Genethon Mega yeast artificial chromosome (YAC) library (Cohen et al, 1993) was screened by PCR, with the following primer pair: HA738 (intron 9 forward), 5′-ATGTTAGAATCATAAGGCCTTTG-3′ and HA691 (exon 10 reverse), 5′-AGGCTGCCGAATAAGGAGAC-3′, nucleotides 1054–1035. The screening DNA pools were provided by Riken, Tsukuba Life Science Center (Ibaragi, Japan). Physical mapping data for markers in the 3p14.2 region were derived from previously published data (Aburatani et al, 1996) and the Whitehead database (internet address: http://www-genome.wi.mit.edu) contig WC-3.10.

### Statistical analysis

The probability of survival (± standard error) was calculated using the product limit method of Kaplan and Meier (Kaplan and Meier, 1958) and compared using the Mantel–Haenszel log rank test. The Fisher exact test was used for the analysis of 2 × 2 tables. The Wilcoxon Mann–Whitney test was used when two groups were compared and Kruskal–Wallis analysis was used for the comparison of several groups.

### Ethical approval

The present study was approved as a multicentre study by the ethics committees of the Uppsala University, Karolinska Institute, Stockholm and the University of Gothenburg.

### RESULTS

### LOH analysis with chromosome 3 markers

DNAs from patients with neuroblastoma ($n = 59$) were subjected to a LOH study using microsatellite markers on different locations on chromosome 3. The markers used and their genetic locations are shown in Figure 1. Nine tumours (15.3%) showed LOH for at least two chromosome 3 markers. Of these, four tumours showed LOH for all informative markers used, indicating that a complete copy of chromosome 3 was lost in the tumour. In contrast, five tumours detected loss of distal 3p markers only, while 3q markers and more proximal 3p markers were retained in two copies. LOH for chromosome 3 was found in neuroblastomas of all the different clinical stages. A particular region on 3p was deleted in all nine tumours. This region could be defined by the deletion patterns in two tumours. One of the tumours (ST102) had a breakpoint between markers D3S1768 and D3S1295. This defines the proximal border of the smallest region of overlap (SRO). The distal border was defined by an interstitial deletion in one tumour (189). The distal deletion breakpoint for this tumour was between markers D3S1286 and D3S1768, and the proximal breakpoint was between markers D3S1766 and D3S1598. Taken together, these data give a smallest region of overlap (SRO) of deletion defined distally by marker D3S1286 and proximally by D3S1295, i.e. 3p25.3–p14.3 (Figures 1 and 2).

### Localization of the FHIT gene relative to the 3p SRO in neuroblastomas

In order to localize the most telomeric part of the FHIT gene, i.e. exon 10, relative to the deletions in the neuroblastomas, we performed STS mapping vs the CEPH Mega YAC library. YACs

**Table 1** Clinical and biological features of the neuroblastoma patients used in the study

| | 3p and 3q LOH | | | | Distal 3p LOH | | | | | No LOH for chromosome 3 or 1 | LOH for 1p but not chromosome 3 |
|---|---|---|---|---|---|---|---|---|---|---|---|
| *n* | 4 | | | | 5 | | | | | 40 | 10 |
| Patient no | 181 | ST107 | 162 | 153 | ST99 | 121 | 174 | ST102 | 189 | | |
| Sex | F | F | M | F | F | M | F | M | M | 20=F, 20=M | 7=F, 3=M |
| Age[a] | 122 | 10 | 0 | 11 | 35 | 72 | 50 | 15 | 30 | 14.5 (0–159), median (range) | 15 (5–52) |
| Stage[b] | 1 | 1 | 4S | 3 | 3 | 2A | 4 | 4 | 4 | 8=1, 8=2, 9=3, 12=4, 3=4S | 10=4 |
| 1p LOH | – | – | – | – | + | + | + | + | + | 40– | 10+ |
| NMA[c] | – | – | – | – | – | – | + | + | + | 34–, 3+, 3 ND | 2–, 8+ |
| Outcome[d] | NED | NED | NED | NED | DOD | DOD | DOD | DOD | DOD | 27 NED, 10 DOD, 3 AWD | 2 NED, 8 DOD |
| Survival[e] | 32+ | 24+ | 52+ | 57+ | 10 | 96 | 6 | 11 | 17 | | |
| Remarks[f] | | | | | | NF1 | | | | | |

[a]Age at diagnosis in completed months; [b]stage 1, 2, 3, 4 and 4S according to INSS; [c]NMA, N-*myc* amplification, positive if > three copies per haploid genome, ND, not done; [d]NED, no evidence of disease, DOD, dead of disease, AWD, alive with disease; [e]Survival in completed months from diagnosis until death or last follow-up (+); [f]NF1 phenotype with homozygous deletion of the NF1 tumour-suppressor gene in the tumour (Martinsson et al, 1997).

*© Cancer Research Campaign 1998*

**Figure 1** Summary of patients displaying LOH for chromosome 3 markers. To the left is a genetic map of markers used in the study. Centimorgan distances are indicated between markers. Identification number, stage and outcome are displayed at the top. N, no evidence of disease; D, dead of disease. The LOH pattern is shown for each patient and marker. (○) No loss of heterozygosity; (●) loss of heterozygosity; –, non-informative marker; blank regions, not done. Areas of deletion are shaded dark grey and areas of no deletion are white; undefined areas are shaded light grey. The smallest region of overlap of deletion (SRO) is indicated to the left

**Figure 2** LOH analysis for chromosome 3 markers of the normal DNA (N) and tumour DNA (T) in patients 162, 181, ST102 and 189. Markers used are indicated on the left, and alleles detected are displayed under each photograph. (**A**) Analyses of patients with favourable outcome; (**B**) analyses of patients with unfavourable outcome positive for FHIT exon 10 were 958-E-3, 958-H-12, 958-E-12, 768-A-7, 944-H-10 and 768-D-2. When comparing our data with those from the region of interest from the Whitehead human physical map project, the following combined order could be obtained for selected markers: 3pter–D31295–D3S1592–D3S1766–(D3S1313–D3S1547)–FHITexon10–D3S1300/FHIT-intron5–D3S1312–D3S1600–3cen.

### Correlation of patterns of chromosome 3 LOH with 1p LOH, N-*myc* amplification, clinical features and prognosis

Two distinct patterns of chromosome 3 aberrations were found in neuroblastomas showing either a complete loss of one copy ($n = 4$) or LOH restricted to distal 3p markers only ($n = 5$). Tumours with these different patterns showed significant clinicobiological characteristics (Table 1). Distal 3p LOH was only detected in tumours with concomitant LOH for 1p (5/15, $P = 0.0006$). Three out of five of these tumours were N-*myc* amplified (vs 0/4, $P = 0.059$), and there was a non-significant trend to more unfavourable clinical stages in these children with distal 3p LOH compared with those with complete chromosome 3 LOH (three out of four of metastatic INSS stage 4 vs 0/4, $P = 0.12$). There were no significant differences with regard to sex. All children with distal 3p LOH were older than the median age at diagnosis (14.5 months), whereas three out of four with total 3 LOH were below one year of age ($P = 0.048$).

All children with a complete loss of one chromosome 3 copy are alive and well 2 years or more after diagnosis, contrasting with the group with distal 3p LOH, in which all have died during follow-up ($P = 0.008$). Survival probability according to Kaplan–Meier in these two groups at 3 years as 100% and 20% (±17.9%) respectively ($P = 0.026$, $\chi^2 = 4.941$, d.f. = 1, Mantel–Haenszel log rank test). Survival probability for children with neuroblastomas without detected chromosome 3 aberrations was 59.3% ± 7.6% between the two groups of children with detected chromosome 3 aberrations ($P = 0.019$, $\chi^2 = 7.956$, d.f. = 2).

As distal 3p LOH was only found in tumours with 1p LOH, we compared subsets of children with tumours showing 1p LOH with and without 3p LOH. LOH for 3p correlated with older age ($P < 0.05$) and was found in non-metastatic tumours (2/5 vs 0/10, $P = 0.095$), but there was no difference in survival probability ($P = 0.78$). All tumours with a complete 3 LOH had a triploid DNA content in tumour cells (data not shown).

### DISCUSSION

Two lines of evidence have indicated that genes on chromosome 3p may be important in the development and/or progression of neuroblastoma tumours. First, we performed a genome-wide scan for loss of heterozygosity (LOH) with polymorphic markers in a subset of different-staged neuroblastomas to get an overview of which chromosomal region, if any, in addition to chromosomal region 1p (Martinsson et al, 1995), would be commonly deleted in our material. Some regions frequently found by other authors to be involved in deletion were not frequently deleted in our neuroblastoma material (Hallstensson et al, 1997). Instead, we found that chromosome 3 loci were deleted at relatively high frequency. Secondly, we performed representational difference analysis (RDA) in order to obtain clones selectively lost in neuroblastoma tumours

© Cancer Research Campaign 1998

(Hallstensson et al, 1997). No clones homozygously lost were obtained in the experiments. However, hemizygously deleted clones, i.e. clones resulting from LOH of chromosomal regions, were found. On detailed analysis of these clones, we found that a large fraction mapped to chromosome 3p (Hallstensson et al, 1997). On these grounds, a larger, more detailed study was made in order to evaluate the extent of deletions on chromosome 3 in neuroblastomas.

We found that 15.3% of neuroblastomas in our material displayed 3p LOH (Figures 1 and 2). This is the third most common genetic aberration in our material, after 1p LOH and N-myc amplification (Martinsson et al, 1995). In the genome-wide scan for LOH, we found the 'background level' of LOH in our material to be low (Hallstensson et al, 1997). The smallest region of overlap of chromosome 3 deletions in the tumours was distally defined by marker D3S1286 and proximally defined by marker D3S1295, i.e. 3p25.3–14.3 (Figures 1 and 2). A striking feature in the 3 LOH pattern of the analysed tumours was that two clinically distinct subgroups could be discerned: (1) a group of neuroblastomas with loss of a complete chromosome 3 associated with a favourable outcome; and (2) a group of neuroblastomas with partial 3p loss in which all patients had a poor outcome (Table 1). These data add further to the picture of neuroblastoma as being a disease with large clinical heterogeneity related to specific features on the molecular level (Brodeur, 1995). Thus, favourable tumours prone to spontaneous differentiation and / or regression often show a triploid karyotype without structural abnormalities, suggesting whole chromosomal gains or losses. Indeed, our data show that a subset of favourable tumours had lost one complete chromosome 3 and probably duplicated the other retained allele (Figure 2). On the other hand, unfavourable neuroblastomas are often near-diploid, harbouring structural genetic aberrations presumed to alter the function of oncogenes or tumour-suppressor genes specifically. The present subset of five tumours with distal 3p LOH associated with poor prognosis indicates the presence of an additional tumour-suppressor locus involved in the tumorigenesis of aggressive neuroblastomas. Furthermore, we hypothesize that this subset of unfavourable tumours may be the result of a multistep tumorigenesis involving the loss of tumour suppressors at 1p and 3p in all five cases. In addition, one tumour had lost both copies of the NF1 tumour suppressor (Martinsson et al, 1997) and three other tumours showed amplification of the N-*myc* oncogene.

The results in the present study show the association between specific aberrations of chromosome 3 and prognosis in neuroblastoma, with poor outcome in all children having a tumour with distal 3p LOH. However, based on this limited material, we cannot suggest the analysis of chromosome 3 for prognostic evaluation of individual patients, as there are a number of other well-characterized prognostic indicators useful for risk assessment of neuroblastoma patients (Castleberry et al, 1997). Poor outcome in the present material was significantly associated with recognized risk factors, such as age over 1 year ($P = 0.007$), metastatic stage 4, amplification of N-myc and 1p LOH (all $P < 0.001$ in univariate analysis).

A few cases of chromosome 3 aberrations in neuroblastoma have been reported earlier: one patient with a dup3q syndrome (Maier and Beck, 1992) and one fetus with neuroblastoma that showed a partial dup3q, unbalanced translocation 3;10 (Qureshi et al, 1994). The case in these duplicate syndromes is often that one entire chromosome is duplicated and the other is missing except for one remaining part, in this case 3q. This means that, in dup3q, one copy of 3p is missing and the other is duplicated. This is in agreement with our results. In the tumour DNA of some of our patients showing LOH, the remaining allele gave a stronger signal than it did in the corresponding normal DNA, although the same amount of genomic DNA had been used (for representative analyses see Figure 2). This can indicate that the LOH in these tumours is accompanied by the addition of one or more copies of the retained allele. It has not been possible to perform cytogenetic analysis of these tumours to confirm these data.

Homozygous and heterozygous deletions in this region on chromosome 3 have frequently been detected in a number of different cancers, e.g. lung cancer (Roche et al, 1996) and breast cancer (Buchhagen et al, 1994). Several genes with potential tumour-suppressing activity have been shown to locate to this region. Recently, the FHIT gene, which spans the 3:8 translocation break in a renal cell carcinoma (Ohta et al, 1996), was isolated and cloned. It is known that the polymorphic marker D3S1300 is located in intron 5 within the FHIT gene itself (Ohta et al, 1996). The tumour with the most distal deletion, ST102 (Figure 1), has a deletion of marker D3S1768 as well as more distal markers, while D3S1295 and markers proximal to it have both alleles retained. D3S1295 is clearly distal to D3S1300, indicating that the deletion in ST102 is distal to the FHIT gene. The tumour with the second most distal deletion, 174, has two copies retained for marker D3S1766, while one copy of D3S1295 is lost in the tumour (Figure 1). The FHIT gene is reported to be very large and distributed over at least 500 kb of genomic DNA (Ohta et al, 1996). The most telomeric of the FHIT exons (exon 10) has not to our knowledge been mapped previously. Therefore, its location relative to marker D3S1766 was not known. Using STS mapping to the CEPH Mega YAC library, we showed that the order of the critical markers is 3pter–D3S1766–(D3S1313–D3S1547)–FHITexon10–D3S1300/FHITintron5–3cen. Thus, marker D3S1766, which is retained in tumour 174, is clearly distal to the most distal FHIT exon. Therefore, the deletion in tumour 174 also maps distal to the FHIT gene. The three remaining tumours with distal 3p deletions had LOH patterns indicating that FHIT may be within the deletion. In one case, ST99, the deletion breakpoint is in or proximal to the FHIT gene. The fact that the 3p SRO in neuroblastoma tumours is distal to the FHIT gene may indicate that another gene distal to FHIT is critical for neuroblastoma tumorigenesis. This also pertains to other cancers with 3p aberrations.

In conclusion, we have detected a high incidence of chromosome 3 LOH in neuroblastoma tumours, with a shortest region of overlap of deletions between markers D3S1286 and D3S1295. In at least two tumours, the deletions map distal to the proposed tumour-suppressor gene FHIT located in 3p14.2. Tumours with deletions of parts of 3p only were associated with a poor prognosis, whereas tumours with loss of a complete copy of chromosome 3 had a favourable outcome. A gene on chromosome region 3p, located distal to FHIT, may be involved in neuroblastoma tumorigenesis.

### ACKNOWLEDGEMENTS

We gratefully acknowledge the financial support of the Swedish Cancer Society, the Children's Cancer foundation, the King Gustav V Jubilee Clinic Cancer Research Foundation and the Assar Gabrielsson Foundation.

### REFERENCES

Aburatani H, Stanton VP and Housman DE (1996) High-resolution physical mapping by combined Alu-hybridization/PCR screening: construction of a yeast artificial chromosome map covering 31 centimorgans in 3p21–p14. *Proc Natl Acad Sci USA* **93**: 4474–4479

*© Cancer Research Campaign 1998*

Brodeur GM (1995) Molecular basis for heterogeneity in human neuroblastomas. *Eur J Cancer* **31A**: 505–510

Brodeur GM, Seeger RC, Barret A, Berthold F, Castleberry RP, D'Angio G, DeBernardi B, Evans A, Favot M, Freeman AI, Haase G, Hartmann O, Hayes FA, Helson L, Kemshead L, Lampeit F, Niane J, Ohkawa H, Philippe T, Pinkerton CR, Pritchard J, Sawada T, Siegel S, Smith EI, Tsuchida Y and Voute PA (1988) International criteria for diagnosis, staging, and response to treatment in patients with neuroblastoma. *J Clin Oncol* **6**: 1874–1881

Brodeur GM, Pritchard J, Berthold F, Carlsen NLT, Castel V, Castleberry RP, Debernardi B, Evans AE, Favrot M, Hedborg F, Kaneko M, Kemshead J, Lampert F, Lee REJ, Look AT, Pearson ADJ, Philip T, Roald B, Sawada T, Seeger RC, Tsuchida Y and Voute PA (1993) Revisions of the international criteria for neuroblastoma diagnosis, staging, and response to treatment. *J Clin Oncol* **11**: 1466–1477

Buchhagen DL, Qiu L and Etkind P (1994) Homozygous deletion, rearrangement and hypermethylation implicate chromosome region 3p14.3–3p21.3 in sporadic breast cancer development. *Int J Cancer* **57**: 473–479

Caron H, Vansluis P, Buschman R, Dotanque RP, Maes P, Beks L, Dekraker J, Voute P, Vergnaud G, Westerveld A, Slater R and Versteeg R (1996) Allelic loss of the short arm of chromosome 4 in neuroblastoma suggests a novel tumour suppressor gene locus. *Hum Genet* **97**: 834–837

Castleberry RP, Pritchard J, Ambros P, Berthod F, Brodeur GM, Castel V, Cohn SL, De Bernardi B, Dicks-Mireaux C, Frappaz D, Haase GM, Haber M, Jones DR, Joshi VV, Kaneko M, Kemshead JT, Kogner P, Lee REJ, Matthay KK, Michon JM, Monclair T, Roald BR, Seeger RC, Shaw PJ, Shimada H and Shuster JJ (1997) The international neuroblastoma risk groups (INRG): A preliminary report. *Eur J Cancer* **33**: 2113–2116

Cohen D, Chumakov I and Weissenbach J (1993) A first generation physical map of the human genome. *Nature* **366**: 698–701

Dib C, Fauré S, Fizames C, Samson D, Drouot N, Vignal A, Millasseau P, Marc S, Hazan J, Seboun E, Lathrop M, Gyapay G, Morissette J and Weissenbach J (1996) A comprehensive genetic map of the human genome based on 5264 microsatellites. *Nature* **380**: 152–154

Fong C, Dracopoli NC, White PS, Merrill PT, Griffith RC, Housman DE and Brodeur GM (1989) Loss of heterozygosity for the short arm of chromosome 1 in human neuroblastomas: correlation with N-myc amplification. *Proc Natl Acad Sci USA* **86**: 3753–3757

Hallstensson K, Thulin S, Aburatani H, Hippo Y and Martinsson T (1997) Representational difference analysis and loss of heterozygosity studies detect 3p deletions in neuroblastoma tumors. *Eur J Cancer* **33**: 1966–1970

Kaplan EL and Meier P (1958) Nonparametric estimation from incomplete observations. *J Am Stat Assoc* **53**: 457–481

Maier B and Beck JD (1992) Dup 3(q) syndrome and neuroblastoma. *Eur J Pediatr* **151**: 715–716

Martinsson T, Sjöberg RM, Hedborg F and Kogner P (1995) Deletion of chromosome 1p loci and microsatellite instability in neuroblastomas analyzed with short-tandem repeat polymorphisms. *Cancer Res* **55**: 5681–5686

Martinsson T, Sjöberg RM, Hedborg F and Kogner P (1997) Homozygous deletion of the neurofibromatosis-1 (NF1) gene in the tumor of a patient with neuroblastoma. *Cancer Genet Cytogenet* **95**: 183–189

Ohta M, Inoue H, Cotticelli MG, Kastury K, Baffa R, Palazzo J, Siprashvili Z, Mori M, McCue P, Druck T, Croce CM and Huebner K (1996) The FHIT gene, spanning the chromosome 3p14.2 fragile site and renal carcinoma-associated t(3;8) breakpoint, is abnormal in digestive tract cancers. *Cell* **84**: 587–597

Qureshi F, Jaques SM, Johnson MP, Reichler A and Evans MI (1994) Microscopic neuroblastoma in a fetus with a de novo unbalanced translocation 3;10. *Am J Med Genet* **53**: 24–28

Roche J, Boldog F, Robinson M, Robinson L, Varella-Garcia M, Swanton M, Waggoner B, Fishel R, Franklin W, Gemmill R and Drabkin H (1996) Distinct 3p21.3 deletions in lung cancer and identification of a new human semaphorin. *Oncogene* **12**: 1289–1297

Savelyeva L, Corvi R and Schwab M (1994) Translocation involving 1p and 17q is a recurrent genetic alteration of human neuroblastoma cells. *Am J Hum Genet* **55**: 334–340

Srivatsan EE, Murali V and Seeger RC (1991) Loss of heterozygozity for alleles on chromosomes 11q and 14q in neuroblastoma. *Prog Clin Biol Res* **366**: 91–98

Suzuki T, Yokota J, Mugisihima H, Okabe I, Ookuni M, Sugimura T and Terada M (1989) Frequent loss of heterozygosity on chromosome 14q in neuroblastoma. *Cancer Res* **49**: 1095–1098

Van Roy N, Laureys G, Cheng NC, Willem P, Opdenakker G, Versteeg R and Speleman F (1994) 1;17 translocations and other chromosome 17 rearrangements in human primary neuroblastoma tumors and cell lines. *Gene Chrom Cancer* **10**: 103–114

© Cancer Research Campaign 1998